# Diluting Ionic Liquids with Small Functional Molecules of Polypropylene Carbonate to Boost the Photovoltaic Performance of Perovskite Solar Cells

**DOI:** 10.3390/molecules29246045

**Published:** 2024-12-22

**Authors:** Shuo Yang, Shaohua Chi, Youshuai Qi, Kaiyue Li, Xiang Zhang, Xinru Gao, Lili Yang, Jinghai Yang

**Affiliations:** 1 School of Materials Science and Engineering, Changchun University, Changchun 130022, China; 7youshuai@gmail.com (Y.Q.); lkaiyue29@163.com (K.L.); 18255152281@163.com (X.Z.); 17803864790@163.com (X.G.); 2 Laboratory of Materials Design and Quantum Simulation College of Science, Changchun University, Changchun 130022, China; 3 Changchun Institute of Optics, Fine Mechanics and Physics, Chinese Academy of Sciences, Changchun 130033, China; csh15043400625@163.com (S.C.); jhyang1@jlnu.edu.cn (J.Y.); 4 Key Laboratory of Functional Materials Physics and Chemistry of the Ministry of Education, Jilin Normal University, Changchun 130103, China; llyang1980@126.com

**Keywords:** perovskite solar cells, ionic liquids, photovoltaic performance, synergistic effect

## Abstract

It is necessary to overcome the relatively low conductivity of ionic liquids (ILs) caused by steric hindrance effects to improve their ability to passivate defects and inhibit ion migration to boost the photovoltaic performance of perovskite solar cells (PSCs). Herein, we designed and prepared a kind of low-concentration 1-butyl-3-methylimidazolium tetrafluoroborate (BMIMBF_4_) diluted with propylene carbonate (PC) via an ultrasonic technique (PC/IL). The decrease in the decomposition temperature related to the IL part and the increase in the sublimation temperature related to the PC part facilitated the use of PC/IL to effectively delay the crystallization process and passivate the defects in multiple ways to obtain high-quality perovskite films. Moreover, the increased conductivity of PC/IL and the more matched band alignment accelerated electron transport and collection. Finally, the MAPbI_3_- and CsMAFA-based PSCs achieved PCE values of 20.87% and 23.29%, respectively, and their stabilities were greatly improved. This work provides a promising approach to optimizing ILs to achieve multiple functions and boost the performance of PSCs.

## 1. Introduction

Perovskite solar cells (PSCs) have achieved a certified power conversion efficiency (PCE) of more than 26%, which can be ascribed to the advantages of organic-inorganic lead halide perovskite such as high absorption ability, suitable and tunable bandgap, micrometer-scale carrier diffusion length, and cost-effective solution fabrication process [1,2,3,4,5,6,7,8,9,10,11,12]. To date, the greatest barrier to PSC development is still a bottleneck, limiting the commercialization process for PSCs [13,14,15]. Due to low-temperature solution processing, numerous unusual defects can be found in polycrystalline perovskite grain boundaries and surfaces, including iodine ion vacancies (V_I_^−^) and MA ion vacancies (V_MA_^+^), along with deep-level defects such as uncoordinated Pb^2+^, further aggravating the long-term stability and non-radiative recombination [16,17,18,19,20,21,22,23,24].

The utilization of ionic liquids (ILs) has been widely employed to address the above issues to achieve a higher photovoltaic performance due to their ability to regulate crystal nucleation, control crystal growth, and inhibit ion migration [25,26,27,28,29,30,31,32,33,34,35,36]. For example, Snaith et al. demonstrated that 1-butyl-3-methylimidazolium tetrafluoroborate (BMIMBF_4_) can inhibit ion migration, which enhances the long-term stability of devices [37]. Tian et al. further introduced 1-ethyl-3-methylimidazolium tetrafluoroborate (EMIMBF_4_) ILs into cesium lead triiodide perovskite to enhance its long-term stability [38]. Although directly introducing these larger-size ILs to perovskite precursor solutions can usually greatly improve the stability of PSCs, it cannot lead to the necessary improvement in PCE, or, even worse, it can somewhat reduce PCE. This is because ILs remain in perovskite films even after the annealing process due to their low volatility [39,40,41]. On the one hand, the residual cations of ILs randomly aggregate within the perovskite and react with PbI_2_ to form a supramolecular complex, potentially resulting in low conductivity [42]. On the other hand, the residue of the polar anions in the ILs at the interface may hinder charge transfer due to their undesired polarization [38]. Therefore, regulating the properties of ILs themselves is necessary for applying them in perovskite-based on their own physical and chemical properties [31,32,42]. For instance, we previously introduced BMIMBF_4_ and carbon quantum dots (CQDs) synergistically into the MAPbI_3_ layer [43]. The CQDs at the grain boundaries provided a pathway for charge carrier transport, thus resolving the problem of lower conductivity to achieve relatively higher PCE and stability at the same time in IL-based PSCs [43]. To date, exploring an effective strategy to overcome the drawbacks of ILs and make them more suitable for PSCs is still an open challenge.

It is worth noting that the interactions between the cations and anions in ILs and their overall ionic activity are highly dependent on the viscosity and ionic conductivity of ILs [44,45]. We also found that organic solvents were used to enhance the ion conductivity and reduce the viscosity of ILs to improve electrolyte performance in the field of lithium-ion batteries. For instance, 1-ethyl-3-methylimidazolium trifluoromethanesulfonimide was blended with ethylene carbonate (EC) and diethyl carbonate to prepare an electrolyte solution. Due to the interaction between ILs and organic solvents, the ion conductivity was increased, and the viscosity was reduced, which effectively improved the electrochemical performance of the lithium-ion batteries [46]. Similarly, N-methyl-N-propylpiperidinium bis(trifluoromethanesulfonyl)imide was mixed with EC and triethyl phosphate and exhibited characteristics such as higher capacitance, lower viscosity, and increased ion conductivity [47]. Obviously, introducing organic molecules into ILs allows for the adjustment of the latter’s viscosity, thereby modulating the activity of ions. Meanwhile, suitable organic molecules with multiple functional groups will help improve the defect passivation effect for perovskite. Based on these promising advantages, a more reliable and effective optimization strategy based on ILs can be provided, which has excellent potential to help obtain highly efficient and stable PSCs.

Propylene carbonate (PC) possesses the properties of high conductivity and chemical stability, which is why it is widely used in electrolyte solutions as a nontoxic and cheap organic solvent [48,49,50]. It is also a small-molecule Lewis base with permanent dipoles, which is beneficial for passivating the defects in perovskite films [51,52,53,54]. Thus, in this work, to combine the advantages of both PC and ILs, we employed an ultrasound technique to modify the commonly used ILs of BMIMBF_4_ with different amounts of PC to find a brand new additive material, PC/IL and then used it to modify perovskite films to achieve the desired regulation effect on PSCs. The interaction between the PC and ILs was first revealed. The positive effects of PC/IL on the crystal growth process of perovskite films, charge transfers, and photovoltaic performance were investigated in detail.

## 2. Results and Discussion

**Figure 1:**
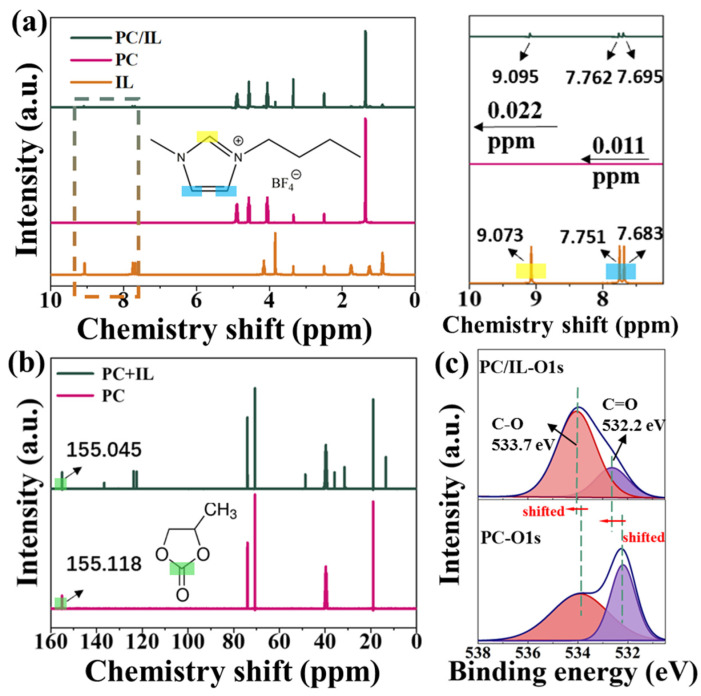
(**a**) ^1^H NMR spectra of PC, IL, and PC/IL, and enlarged views of the areas framed by dashes. The yellow boxes correspond to the hydrogen atoms in N–CH=N, and the blue boxes correspond to the hydrogen atoms in–CH=CH–. (**b**) ^13^C NMR spectra of IL and PC/IL. The green boxes correspond to the carbon atoms in C=O. (**c**) XPS spectra of O 1s orbit of PC and PC/IL.

The interaction between BMIMBF_4_ (named IL for convenience) and PC in the PC/IL was first investigated. Figure 1a shows the ^1^H NMR spectra of IL, PC, and PC/IL in DMSO-d6 and enlarged views of the areas framed by dashes in the full spectra. The highlighted bars in the spectra represent the peaks of hydrogen atoms in IL, aligning with the color-coded atoms in the molecular structure of BMIMBF_4_ [55]. It can be observed that when PC is added to ILs, the peak originating from the cationic proton of ILs shifts significantly downfield. This might be ascribed to the formation of hydrogen bonds between PC and IL, which reduces the electron density around the IL cation. By contrast, it is worth noting that the ^1^H signal originating from the PC does not show significant changes. Therefore, ^13^C NMR tests were further performed on PC and PC/IL to identify the binding sites of hydrogen bonds on PC. As shown in Figure 1b, when PC is introduced into IL, the ^13^C NMR peak of PC marked in green shifts to a higher field, indicating that the interactions between PC and IL primarily occur between the carbonyl groups in PC and the hydrogen in IL cations. Among these interactions, the one that occurred with the H atom (N–CH=N) corresponding to the peak of δ = 9.073 ppm in IL is the strongest since the δ shift is relatively larger, suggesting that this H atom is more active than the others. Notably, the shift direction of δ for the H proton in the IL cation and the shift direction of δ for the carbonyl carbon in PC are opposite, indicating the formation of C–H…O (similar to hydrogen bonds). Since the carbon atom in the imidazole ring of ILs is located between two nitrogen atoms, each carbon atom carries a part of the positive charge (δ+ C–H), making it easier to interact with the carbonyl oxygen (C=O δ−) with strong electronegativity, thus exhibiting partial hydrogen bond properties. As illustrated in the X-ray photoelectron spectra (XPS) shown in Figure 1c, the binding energy of O 1s of PC/IL shifted to a higher binding energy compared to that of PC, further confirming the formation of C–H…O bonds [56].

**Figure 2:**
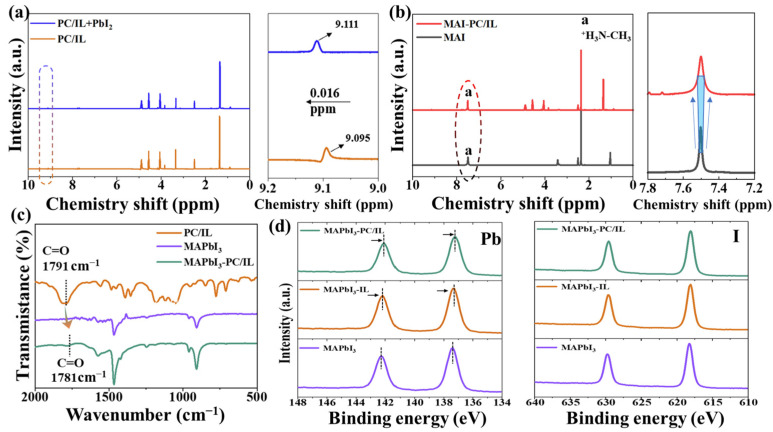
(**a**) ^1^H NMR spectra of PC/IL with/without PbI_2_. The peaks marked by the dashed circle were enlarged and shown on the right side. (**b**) ^1^H NMR spectra of PC/IL with/without MAI and an enlarged image at 7.5 ppm. The peaks marked by the dashed circle were enlarged and shown on the right side. The arrow and blue box indicate that the width of the peak has turned broader. (**c**) FTIR spectra of PC/IL, MAPbI_3_, and MAPbI_3_-PC/IL films. (**d**) XPS spectra of the Pb 4f and I 3d core level lines from MAPbI_3_, MAPbI_3_-IL, and MAPbI_3_-PC/IL films.

The interaction between PC/IL and perovskite precursor was then investigated with ^1^H NMR spectra. As shown in Figure 2a, regarding the mixture of PC/IL and PbI_2_ solution, the hydrogen signal of the imidazole group shifted to the lower field, demonstrating that the coordination occurs between N in the imidazole group and Pb^2+^. Figure 2b presents the ^1^H NMR spectra of MAI with or without PC/IL in DMSO-d6. After the introduction of PC/IL, the width of the H signal originating from NH_3_^+^ is increased, indicating that hydrogen bonding (N-H∙∙∙F) forms between the -F in PC/IL and NH_3_^+^ of MAI. The FTIR spectra of PC/IL, MAPbI_3_, and MAPbI_3_-PC/IL films are shown in Figure 2c. The C=O vibration peak in the PC/IL appears at 1791 cm^−1^. For MAPbI_3_-PC/IL, it shifts to 1781 cm^−1^, which might be attributed to the electronic delocalization caused by the formation of C=O•PbI_2_ intermediate complex adduct, indicating a strong coordination interaction between C=O and Pb^2+^. Proof of this is provided in Figure 2d, which displays the Pb 4f XPS spectra of the control, MAPbI_3_-IL, and MAPbI_3_-PC/IL films. For the control, two main peaks located at 142.3 eV and 137.4 eV can be ascribed to the Pb 4f5/2 and Pb 4f7/2 core lines, respectively. Regarding the MAPbI_3_-IL and MAPbI_3_-PC/IL films, these peaks shift toward lower binding energy, further proving that both ILs and PC/IL can interact with uncoordinated Pb^2+^. Interestingly, the peak shifts of MAPbI_3_-PC/IL are slightly larger than that of MAPbI_3_-IL due to the presence of C=O, which can jointly passivate uncoordinated Pb^2+^ with imidazole groups. Furthermore, no obvious shift occurs in the I 3d XPS spectra, indicating that PC/IL does not interact with I ions. All of these results support the above discussion of FTIR and ^1^H NMR data.

**Figure 3:**
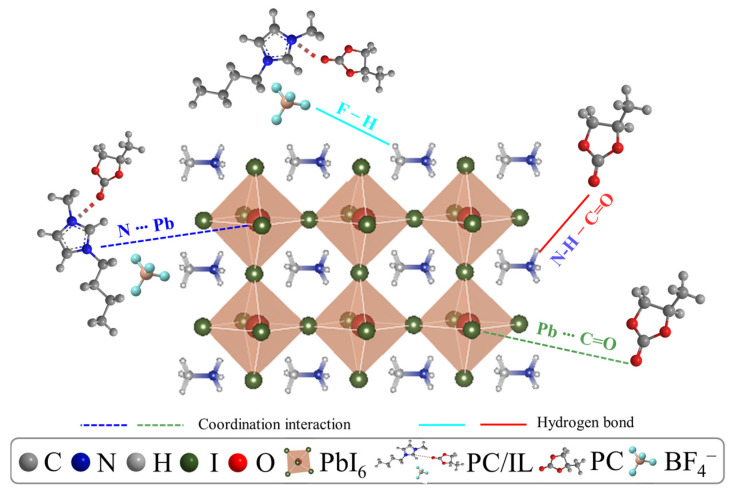
Possible interactions between PC/IL and perovskite precursors.

Based on the above discussion, the possible interactions between PC/IL and perovskite precursors are shown in Figure 3. Regarding the PC/IL materials, due to the high ratio between PC and IL during the preparation process, all BMIM^+^ molecules are combined with PC, but -BF_4_^−^ and some PC molecules exist dissociatively in the PC/IL material. Thus, three parts, namely, PC, BMIM^+^/PC, and –BF_4_^−^, contribute to the interaction between PC/IL and perovskite precursors. Both C=O and N with lone pair electrons in the cationic imidazole group in the PC/IL can coordinate with Pb^2+^. Both C=O and -F in BF_4_^−^ anion can entrap MA^+^ by establishing hydrogen bonds, preventing its escape from the lattices throughout the fabrication and annealing processes. Obviously, multi-passivation effects can be achieved with PC/IL modification, which is beneficial for achieving perovskite films with high crystal quality.

**Figure 4:**
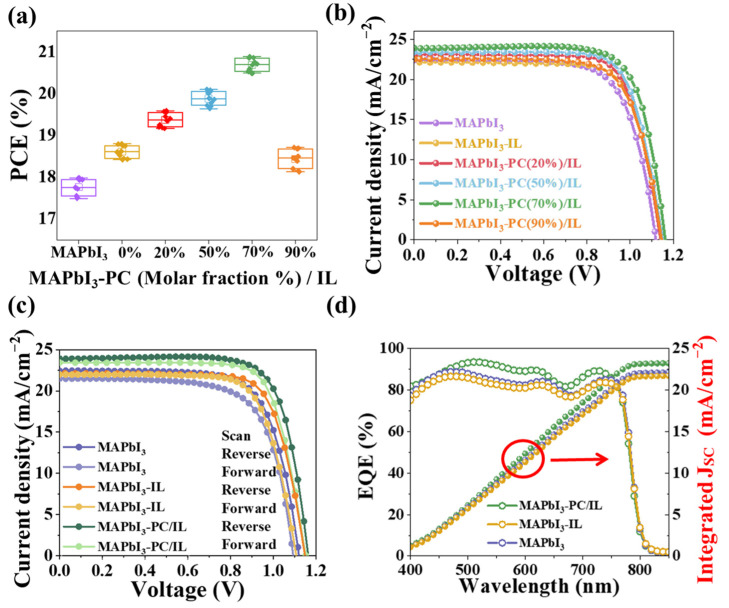
(**a**) Statistical distribution of PCE of the pristine devices (purple) and PSCs based on PC(x%)/IL modification (x = 0% (yellow), 20% (red), 50% (blue), 70% (green), and 90% (orange)). (**b**) J–V curves of MAPbI_3_-based PSCs with PC(x%)/IL modification. (**c**) J–V curves of champion MAPbI_3_, MAPbI_3_-IL, and MAPbI_3_-PC/IL-based PSCs with reverse and forward scans. (**d**) EQE spectra and integrated current curves (marked by the red color circles) for MAPbI_3_, MAPbI_3_-IL, and MAPbI_3_-PC/IL PSCs.

A series of PSCs with the architecture of ITO/SnO_2_/MAPbI_3_/Spiro-OMeTAD/Ag via PC(x%)/IL (x = 0%, 20%, 50%, 70%, and 90%) modification were fabricated to evaluate the dependence of the photovoltaic performance on the proportion of PC; they were named MAPbI_3_, MAPbI_3_-IL, MAPbI_3_-PC (20%)/IL, MAPbI_3_-PC (50%)/IL, MAPbI_3_-PC (70%)/IL and MAPbI_3_-PC (90%)/IL, respectively. The statistical distributions of PCE for these PSCs are shown in Figure 4a. The corresponding optimized J–V curves and photovoltaic parameters are shown in Figure 4b and Table S1, respectively. Obviously, the MAPbI_3_-PC (70%)/IL PSCs achieve the highest PCE, which was then used in the following part to reveal the effects of PC/IL on the photovoltaic performance of PSCs and accordingly named MAPbI_3_-PC/IL.

Figure 4c shows the J–V curves of the champion MAPbI_3_, MAPbI_3_-IL, and MAPbI_3_-PC/IL PSCs with reverse and forward scans, and the corresponding photovoltaic parameters are summarized in Table 1. The MAPbI_3_-PC/IL PSCs achieved a PCE of 20.89%, which is much higher than the values of 17.98% and 18.80% for the MAPbI_3_ and MAPbI_3_-IL PSCs, respectively. Meanwhile, it can be seen that J_SC_, V_OC_, and FF all increased accordingly, and the hysteresis was reduced as well. Figure 4d shows the corresponding external quantum efficiency (EQE) curves.

The MAPbI_3_-PC/IL PSCs exhibit a noticeable enhancement in light response in the range of 500~750 nm, possibly due to the improved crystal quality leading to a reduction in non-radiative recombination [57,58,59]. The integrated Jsc was 22.34 mA cm^−2^, 21.95 mA cm^−2^, and 23.45 mA cm^−2^ for MAPbI_3_, MAPbI_3_-IL, and MAPbI_3_-PC/IL PSCs, respectively, which is consistent with the Jsc results obtained from the J–V curves.

**Figure 5:**
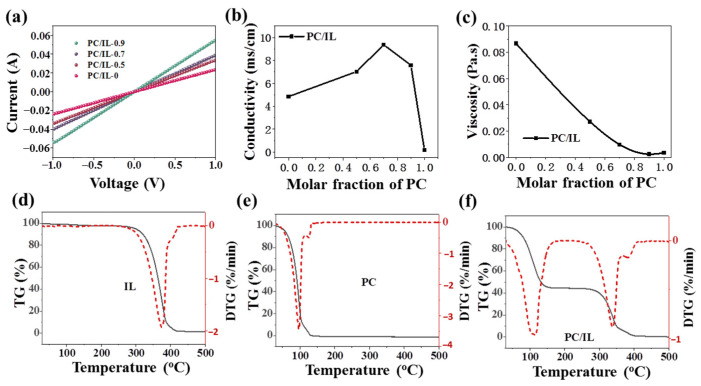
(**a**) Current/voltage (I-V) characteristics of devices (ITO/PC/IL/Ag with different molar fractions of PC: 0%, 50%, 70%, and 90%) under dark conditions. (**b**) Electrical conductivity of PC/IL via the molar fraction of PC at 24 °C. (**c**) The viscosity of PC/IL via the molar fraction of PC. Thermogravimetric analysis (TGA) curves of (**d**) IL, (**e**) PC, and (**f**) PC (70%)/IL in N_2_ atmosphere.

To explore the reasons for the improvement in device performance, we further studied the unique properties of PC/IL composites. Figure 5a shows the current/voltage (I-V) characteristics of the devices (ITO/PC/IL/Ag) with different molar fractions of PC under dark conditions. With the increase in the molar fraction of PC, the conductivity of the PC/IL films significantly increases. This can be attributed to the formation of C–H...O bonds, which can facilitate the charge transfer within the molecule. Accordingly, as shown in Figure 5b and Supplementary Figure S1, the ionic conductivity of PC/IL increases with the increase in the molar fraction of PC as well. When the PC fraction is 0.7, the ionic conductivity increases from approximately 4.85 mS/cm of IL to 9.37 mS/cm. This variation tendency is similar to previous results for ILs mixed with non-ionic molecular compounds (such as acetonitrile, methanol, or carboxylic acids) [60,61,62]. Furthermore, as shown in Figure 5c, the solution viscosity also gradually decreases with the increase in PC content. Both conductivity and viscosity exhibit a consistent tendency for variation in line with Walden’s rule.

Figure 5d–f display the TG–DTG curves of ILs, PC, and PC/IL. The mass loss rate of ILs reaches its maximum at 380 °C, confirming its high thermal stability [63,64,65]. Meanwhile, due to its volatility, PC exhibits a mass loss of about 10% at 60 °C, and the maximum loss rate can be found with endothermic peaks at 97 °C. In contrast, the PC/IL composite exhibits two major weight loss stages accompanied by two strong endothermic peaks at 111 °C and 338 °C, corresponding to the related part of PC and ILs, respectively. Obviously, the PC/IL containing 70% PC shows a mass loss of about zero at 60 °C. The decrease in the decomposition temperature related to IL and the increase in the sublimation temperature related to PC in PC/IL can be attributed to the interactions between PC and IL. This change in PC/IL is beneficial for the two-step annealing process for preparing perovskite films. Specifically, during the initial low-temperature annealing process at 60 °C, almost no additive volatilization occurs in the perovskite film. Figure S2 provides photo images of MAPbI_3_ and MAPbI_3_-PC/IL films dependent on the post-annealing time at 60 °C. The MAPbI_3_ film quickly changes from yellow to brown after heat treatment for 20 s. In contrast, the MAPbI_3_-PC/IL film begins to change color after heat treatment for 1 min. This significantly slower color change phenomenon demonstrates that PC/IL induces a delay in the crystallization of the film due to the multiple interactions between PC/IL and perovskite, which is constructive for forming continuous, smooth, and dense perovskite films.

**Figure 6:**
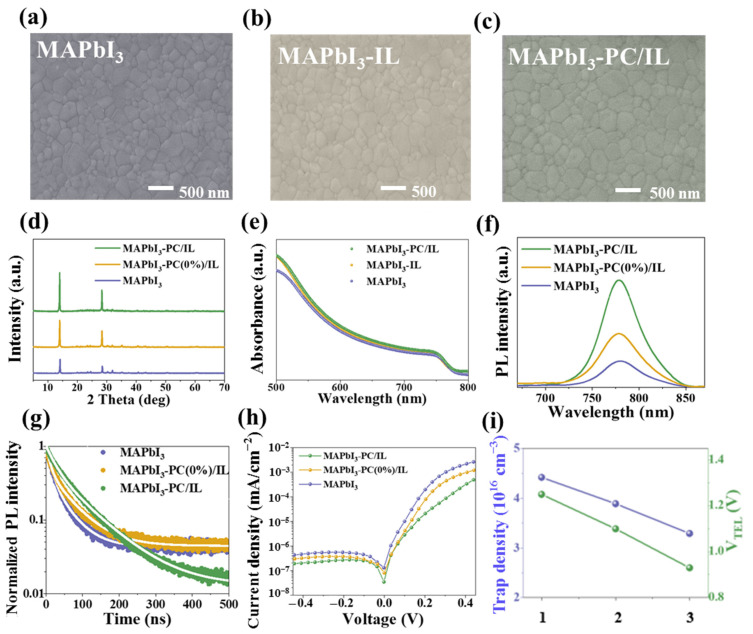
(**a**–**c**) SEM images and (**d**) XRD patterns of MAPbI_3_, MAPbI_3_-IL, and MAPbI_3_-PC/IL films deposited on ITO/SnO_2_. (**e**) UV–visible absorption spectra, (**f**) PL, and (**g**) TRPL spectra of corresponding films deposited on glass substrates. (**h**) Dark I–V curves for MAPbI_3_, MAPbI_3_-IL, and MAPbI_3_-PC/IL PSCs. (**i**) Trap density of corresponding films (1, MAPbI_3_; 2, MAPbI_3_-IL; and 3, MAPbI_3_-PC/IL) obtained from the SCLC method.

Figure 6a–c and Figure S3 display the SEM images and corresponding grain size distributions of the MAPbI_3_, MAPbI_3_-IL, and MAPbI_3_-PC/IL films deposited on ITO/SnO_2_. Compared to the MAPbI_3_ and MAPbI_3_-IL films, a denser and more uniform morphology can be achieved in the MAPbI_3_-PC/IL film with increased grain size. In terms of the crystallization delay caused by PC/IL, we can deduce that the PC/IL actually contributes to the Ostwald ripening effect, promotes mass transfer, and increases the grain size of perovskite crystals [66,67,68]. Figure 6d further displays the X-ray diffraction (XRD) patterns of MAPbI_3_, MAPbI_3_-IL, and MAPbI_3_-PC/IL films. The diffraction intensities of both (110) and (220) peaks—located at 14.4° and at 28.7°, respectively—gradually increase, further proving the positive modulation of PC/IL for improving the crystallinity of MAPbI_3_-PC/IL films [69]. Accordingly, the enhanced absorption intensity across the entire region of MAPbI_3_-PC/IL films can be found in Figure 6e. No obvious change occurs in the band gap deduced from the Tauc plot (Figure S4). The PL and TRPL spectra of MAPbI_3_, MAPbI_3_-IL, and MAPbI_3_-PC/IL films deposited on glass are shown in Figure 6f,g. The fitting parameter for Table S2 and the enhanced emission intensity and extended average lifetime can be observed clearly. All these results are reasonably consistent due to the improved crystal quality, which is the main reason for the improved Jsc presented in Figure 4 [70,71].

The dark I–V curves of MAPbI_3_, MAPbI_3_-IL, and MAPbI_3_-PC/IL PSCs in Figure 6h exhibit the gradual suppression of dark current leakage, indicating that the introduction of PC/IL reduces the defects and inhibits the carrier recombination [72]. To quantitatively analyze the defect density of perovskite films, the space charge limited current (SCLC) curves of pure perovskite devices (ITO/MAPbI_3_, MAPbI_3_-IL, or MAPbI_3_-PC/IL/Ag) are as shown in Figure S5. The defect density can be calculated using the following equation [73]: V_TFL_ = qn_t_L^2^/2εε_0_, where V_TFL_ is the trap-filled limit voltage, q is the electric charge, n_t_ is the defect density, L is the thickness of the perovskite layer (500 nm), ε is the dielectric constant of the perovskite, and ε_0_ is the vacuum permittivity. The as-determined V_TFL_ was decreased from 1.25 V for the MAPbI_3_ film to 1.09 V for the MAPbI_3_-IL film and 0.93 V for the MAPbI_3_-PC/IL film (Figure 6i). Accordingly, the n_t_ was reduced from 4.42 × 10^16^ cm^−3^ and 3.89 × 10^16^ cm^−3^ to 3.29 × 10^16^ cm^−3^. The reduced defects in the MAPbI_3_-PC/IL film confirm the effectiveness of PC/IL in crystal quality modulation and defect passivation.

To further confirm the suppression of carrier recombination within PSCs, the electrical impedance spectra are shown in Figure S6. The incomplete semicircles at low frequencies can extract recombination resistances (Rrec) to represent the carrier recombination process. The arcs at high frequency can extract transport resistance (Rct) to represent carrier transport features in the bulk layer. The MAPbI_3_-PC/IL PSCs show a larger Rrec and lower transport resistance (Rct) than MAPbI_3_ and MAPbI_3_-IL PSCs (Table S3), indicating lower recombination loss and a faster charge/carrier transfer process, which is in good agreement with the reduced defects proved by the SCLC results. The transient photovoltage (TPV) was further determined to gain a deeper understanding of the transient recombination dynamics. As shown in Figure S7, the photovoltage decay time of MAPbI_3_-PC/IL PSCs is the longest, providing further evidence of the superior suppression effect on carrier recombination in MAPbI_3_-PC/IL PSCs.

**Figure 7:**
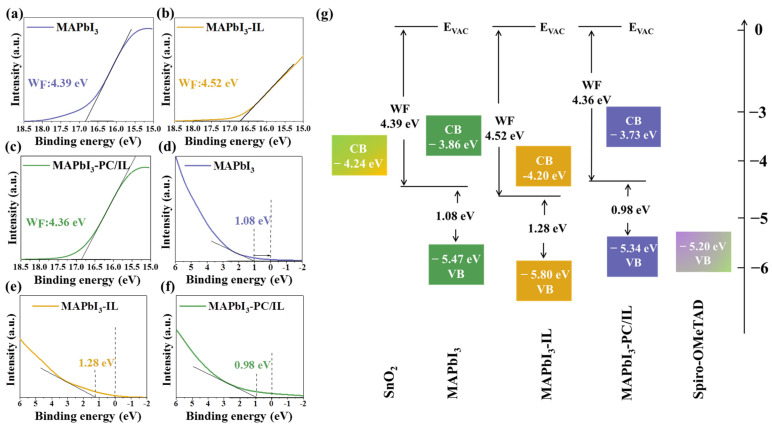
Secondary electron cut-off region of UPS spectra of (**a**) MAPbI_3_, (**b**) MAPbI_3_-IL, and (**c**) MAPbI_3_-PC/IL films. Valence band region of the UPS spectra of (**d**) MAPbI_3_, (**e**) MAPbI_3_-IL, and (**f**) MAPbI_3_-PC/IL films. (**g**) Energy band diagram of MAPbI_3_, MAPbI_3_-IL, and MAPbI_3_-PC/IL-based PSCs.

Ultraviolet photoelectron spectroscopy (UPS) was performed to evaluate the band alignment variation caused by the introduction of PC/IL (Figure 7). As shown in Figure 7a–f, the conduction band minimum (CBM) values for MAPbI_3_, MAPbI_3_-IL, and MAPbI_3_-PC/IL were determined to be −3.87, −4.20, and −3.84 eV, respectively. The valence band maximum (VBM) values for MAPbI_3_, MAPbI_3_-IL, and MAPbI_3_-PC/IL were −5.47, −5.80, and −5.34 eV, respectively. The band alignment of each PSC is illustrated in Figure 7g. Evidently, the incorporation of IL leads to unmatched energy levels in PSCs compared to the control device, which can be ascribed to the existence of BF_4_^−^ anions with high electronegativity at the interface between MAPbI_3_-IL films [38,43]. However, when PC/IL is introduced, the change in the band alignment of the perovskite film is beneficial for charge transfer and collection due to the possible depolarization phenomenon on the film surface, which alters the dipole effect on the film surface [74]. To further confirm this, we used the F1s XPS spectra of the MAPbI_3_, MAPbI_3_-IL, and MAPbI_3_-PC/IL films, shown in Figure S8. Compared to the MAPbI_3_-IL films, the F signal was strongly reduced in the MAPbI_3_-PC/IL films due to the reduced viscosity of PC/IL mentioned previously [75,76]. The 70% mole ratio of PC within PC/IL changes not only the activity of ILs but also the distribution of BF_4_^−^ anions on the surface of the perovskite film, which effectively alleviates the polar strength and then moves the energy level to the desired place.

To assess the repeatability of devices, the J-V characteristics of a series of MAPbI_3_, MAPbI_3_-IL, and MAPbI_3_-PC/IL-based PSCs are shown in Figure S9. All devices exhibited small standard deviations, indicating that the PC/IL-modulating effects on PSCs are highly reproducible. The corresponding photovoltaic parameters of the champion devices are summarized in Table S4. The MAPbI_3_-IL PSCs exhibit significant improvements in V_OC_ and FF due to their improved crystal quality and reduced trap sites [77]. However, the formation of supramolecular complexes between ILs and PbI_2_ increases the spatial hindrance in the device, leading to a decrease in J_SC_ [42]. By contrast, MAPbI_3_-PC/IL PSCs demonstrate remarkable improvements in J_SC_, V_OC_, and FF, surpassing the performance of MAPbI_3_-IL PSCs, demonstrating that diluting ILs with PC is necessary to boost the photovoltaic performance of PSCs. Promisingly, to the best of our knowledge, a PCE of 20.89% achieved in MAPbI_3_-PC/IL PSCs is one of the few PCE values approaching 21% for n-i-p planar IL-based MAPbI_3_ PSCs (Table S5).

**Figure 8:**
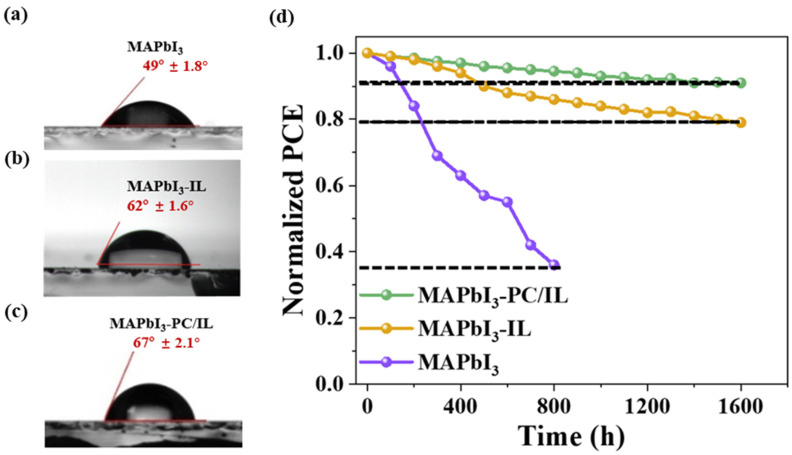
Water contact angles of (**a**) MAPbI_3_, (**b**) MAPbI_3_-IL, and (**c**) MAPbI_3_-PC/IL films. (**d**) PCE variation curves dependent on the storage time (25 °C, RH = 20–30%) of MAPbI_3_, MAPbI_3_-IL, and MAPbI_3_-PC/IL PSCs. The dashed line is used to point out the final normalized PCE value.

Ultimately, we focused on assessing the impact of PC/IL on the stability of PSCs. Our initial exploration involved examining the hydrophobicity/hydrophilicity of the top surface of MAPbI_3_, MAPbI_3_-IL, and MAPbI_3_-PC/IL films through water contact angle measurements. As depicted in Figure 8, the water-droplet’s contact angle exhibited an increase from 49° in the control film to 62° and 67° in the MAPbI_3_-IL and MAPbI_3_-PC/IL films, respectively. This indicates that the introduction of PC/IL results in a more hydrophobic surface, effectively repelling moisture and enhancing the stability of perovskite films in the surrounding environment [78]. Subsequently, the stabilities of MAPbI_3_ PSCs, MAPbI_3_-IL PSCs, and MAPbI_3_-PC/IL PSCs were examined in an ambient atmosphere (25 °C, RH = 20–30%). The corresponding variation curves of PCE for 1600 h are presented in Figure 8c. It shows that MAPbI_3_-PC/IL devices can still retain ~90% of the value of their initial PCE after 1600 h, while MAPbI_3_ devices rapidly decrease to 34% after 800 h. Thus, introducing PC/IL effectively enhances both the PCE and the stability of PSCs.

To verify the universality of the PC/IL synergy modification strategy, mixed cation perovskite (CsFAMA)-based PSCs with an n-i-p structure were fabricated. The J–V curves for the CsFAMA and CsFAMA-PC/IL-based PSCs are shown in Figure S10, and the corresponding photovoltaic parameters with the best performance are given in Table S6. Compared to the CsFAMA-based device, the PC/IL-modified device exhibits better photovoltaic performance; V_OC_, J_SC_, and FF increase at the same time; and the highest PCE of 23.29% has been achieved, which also exhibits superiority among previous similar reports (Table S7).

## 3. Materials and Methods

The materials used in this work and the fabrication technique for MAPbI_3_ PSCs and CsFAMA-based PSCs are similar to those in our previous works [43,54]. The different parts are described below: PC and BMIMBF_4_ were purchased from Aladdin Shanghai, China. The PC was added to the BMIMBF_4_ with different volume ratios (0, 50%, 70%, 90%, and 100%), and they were treated under ultrasonic conditions at room temperature for 24 h. The obtained materials were named as PC(x%)/IL. During the fabrication of PSCs, 0.4 mol% PC(x%)/IL was incorporated into the perovskite precursors. A detailed description of the method can be found in the Supporting Information.

## 4. Conclusions

ILs diluted with PC in an optimized mole ratio were synthesized and utilized to modify perovskite films. Three positive functions of PC/IL were proven: (1) Defect passivation effects were greatly enhanced by the synergistic action of IL and PC. The C=O bond of the carbonyl group and the N with lone-pair electrons in the imidazole group in the PC/IL can passivate the defects of uncoordinated Pb^2+^. Simultaneously, the BF_4_^¯^ anion can trap MA^+^ by forming hydrogen bonds to suppress ion migration and stabilize the crystal structure. (2) The decrease in the decomposition temperature related to the IL part and the increase in the sublimation temperature related to the PC part facilitate a delay in the crystallization process to obtain high-quality perovskite films. (3) The increased conductivity of PC/IL and optimized energy level alignment accelerate the charge transfer process. As a result, the MAPbI_3_-based PSCs achieved a PCE of 20.87%, and the CsMAFA-based PSCs achieved a PCE of 23.29%. This work not only demonstrates the feasibility of molecular-modified ILs as additives to achieve efficient and stable PSCs but also provides a promising approach for improving the performance of perovskite-based photoelectric devices and next-generation optoelectronic devices [79,80,81,82].

## Figures and Tables

**Table 1:** Photovoltaic parameters and hysteresis index of champion MAPbI_3_, MAPbI_3_-IL, and MAPbI_3_-PC/IL PSCs.

Perovskite	Scan Direction	V_OC_ (V)	J_SC_ (mA cm^−2^)	FF (%)	PCE (%)	HI
MAPbI_3_	Forward	1.09	21.48	70.03	16.42	0.09
	Backward	1.12	22.42	71.74	17.98	
MAPbI_3_-IL	Forward	1.11	21.98	73.22	17.80	0.05
	Backward	1.14	22.14	74.15	18.80	
MAPbI_3_-PC/IL	Forward	1.15	23.42	74.65	20.11	0.04
	Backward	1.16	23.90	75.21	20.89	

## Data Availability

The data supporting this article have been included as part of the ESI.

## Supplementary Materials

The following supporting information can be downloaded at www.mdpi.com/article/10.3390/molecules29246045/s1: The production and analysis of PC/IL, photos, Tauc plots, and defect passivation spectrograms for MAPbI_3_, MAPbI_3_-IL, and MAPbI_3_-PC/IL films, critical J−V curves of PSCs, and the tables of the photovoltaic parameters of PSCs, fitted XPS, and a tabulated summary of statistical data for representative MAPbI_3_-IL and CsFAMA-ILPSCs.
