# Supplementary material for: Diluting Ionic Liquids with Small Functional Molecules of Polypropylene Carbonate to Boost the Photovoltaic Performance of Perovskite Solar Cells
Source: Molecules. 2024 Dec 22;29(24):6045. doi: 10.3390/molecules29246045 (PMC11678742; doi:10.3390/molecules29246045)
Supplement: Supplementary file 1 [file molecules-29-06045-s001.zip › molecules-3364418-supplementary.pdf]

Supporting Information

Diluting Ionic Liquids with Small Functional Molecules of Polypropylene Carbonate to Boost the Photovoltaic Performance of Perovskite Solar Cells

Shuo Yang [1,2,*,†], Shaohua Chi [3,4,†], Youshuai Qi [1,2], Kaiyue Li [1,2], Xiang Zhang [1,2], Xinru Gao [1,2], Lili Yang [4] and Jinghai Yang [3,4]

[1] School of Materials Science and Engineering, Changchun University, Changchun 130022, China; 7youshuai@gmail.com (Y.Q.); lkaiyue29@163.com (K.L.); 18255152281@163.com (X.Z.); 17803864790@163.com (X.G.)

[2] Laboratory of Materials Design and Quantum Simulation College of Science, Changchun University, Changchun 130022, China

[3] Changchun Institute of Optics, Fine Mechanics and Physics, Chinese Academy of Sciences, Changchun 130033, China; csh15043400625@163.com (S.C.); jhyang1@jlnu.edu.cn (J.Y.)

[4] Key Laboratory of Functional Materials Physics and Chemistry of the Ministry of Education, Jilin Normal University, Changchun 130103, China; llyang1980@126.com

* Correspondence: yangs@ccu.edu.cn

† These authors contributed equally to this work.

Experimental sections

Materials: All materials and reagents, including lead iodide (PbI_2), methylammonium iodide (MAI), Methylammonium bromide (MABr), Cesium iodide (CsI), Methylammonium chloride (MACl), lead bromide ($PbBr_2$), SnO_2 (10% aqueous colloidal dispersion), chlorobenzene (CB), Tris(2-(1H-pyrazol-1-yl)-4-tert-butylpyridine) cobalt (III) trifluonimide (FK-209), were utilized without additional purification and were acquired from Sigma-Aldrich. ITO glass substrates, Spiro-OMeTAD, and all anhydrous solvents were obtained from BoronTech (China). PC and $BMIMBF_4$ were purchased from Aladdin Shanghai.

Preparation of perovskite precursor solutions: The $MAPbI_3$ perovskite precursor solution was prepared by mixing 1 M PbI_2 and 1 M MAI in mixed DMF/DMSO solvent system (7:3). Similarly, the CsFAMA perovskite precursor solution was prepared with 7.4 mg of MABr, 18.2 mg of CsI, 20 mg of MACl, 26.5 mg of $PbBr_2$, 217.3 mg of FAI, and 642.9 mg of PbI_2 in 1 ml of a mixture of N, N-dimethylformamide (DMF) and dimethyl sulfoxide (DMSO) in a ratio of 4:1. In the perovskite precursor solution containing ionic liquid (IL), we introduced $BMIMBF_4$ (0.4 mol%). The resulting solutions were blended and filtered (0.45 μm, PTFE) in a glove box before utilization.

Preparation of $MAPbI_3$ device: The ITO glass is cleaned with cleaning agent, deionized water, ethanol, and isopropyl alcohol, and then dried with nitrogen gas.

Conduct a 600 s ultraviolet ozone treatment. The SnO_2 was spin-coated onto the ITO substrates, and anneal at room temperature for 30 min at 150°C. Then, the perovskite precursor was spin-coated at a speed of 4500 revolutions per minute for 35 seconds. Then, films were annealed at 60°C for 5 min in an N_2 atmosphere, followed by additional annealing at 100°C for 10 min. The film prepared using IL was referred to as "$MAPbI_3$-IL," while the film prepared using PC/IL is called "$MAPbI_3$-PC/IL." Subsequently, 70 μL of Spiro-OMeTAD was deposited on the annealed perovskite film using a spin-coating method, with a spin-coating duration of 35 s at a speed of 3000 rpm. Finally, under vacuum conditions, 100 nm Ag electrode was deposited using a thermal evaporation technique.

Preparation of CsFAMA device: The ITO glass substrates were subjected to a series of cleaning steps, including sequential sonication in detergent, deionized water, ethanol, isopropanol, and then dried with dry nitrogen, followed by a 20 min UV-ozone treatment. The SnO_2 precursor solution was formulated by stirring a SnO_2 colloidal dispersion in deionized water for 12 h at a volume ratio of 1:3. Subsequently, a layer of SnO_2 electron transport was coated on the ITO glass and spun at a speed of 4000 rpm. Following that, annealing treatment was conducted for 30 min at 150°C in ambient air. The ITO/SnO_2 substrates were then transferred into a glove box filled with nitrogen for perovskite film deposition. To create the CsFAMA perovskite film, a precursor solution (90 μL) was spin-coated at 5000 rpm for 30 s. The film underwent annealing at 100 °C for 1 h. The film, prepared from PC/IL and named "CsFAMA-PC/IL," underwent annealing at 100°C for 1 h. Then 75 μL of Spiro-OMeTAD was

spin-coated onto CsFAMA films at 4000 rpm for 30 s. Successfully deposited Ag electrodes of 100 nm using the thermal evaporation method under vacuum conditions.

Characterization

The scanning electron microscope (SEM) images was taken using a Hitachi S-4800. The crystal structure of the $MAPbI_3$ films was carried out by X-ray power diffraction (XRD) (Japan Rigaku D/max-ga X-ray diffractometer) erica) using Cu Kα (λ = 0.15406 nm) source. The steady-state photoluminescence spectra (PL) were collected from FL-1000 fluorescence spectrometer (Horiba Jobin Yvon Fluorolog-3) with a 405 nm CW laser excitation source. The time-resolved PL measurements (TRPL) measurements were performed on a Horiba Jobin Yvon Fluorolog-3 fluorescence lifetime spectrometer d by using an excitation wavelength of 385nm. The electrical impedance spectroscopy (EIS) was performed by using the electrochemical workstation (CHI660C, Chen Hua, China) with the frequency range from 10 Hz to 0.1MHz in the dark. The samples for XPS and UPS measurements were deposited onto the Si substrate. The X-ray photoelectron spectroscopy (XPS) data were obtained by an ESCALab220i-XL electron spectrometer from VG Scientific. The ultraviolet photoemission spectroscopy (UPS) data were taken under UHV with a helium discharge lamp (hν = 21.22 eV). The transient photovoltage spectrum (TPV) was measured by CEL-TPV2000. Liquid-State ^1H NMR was measured with standardized parameter sets from Bruker Topspin version 2.1 (AV400) and 3.0 (AV500). The Fourier transform infrared (FTIR) spectrum was collected on a Nicolet iS50 FTIR spectrometer. The main corresponding setup consisted of

perovskite, mica-flakes. J-V characterizations was carried out was carried out under AM 1.5 G simulated sunlight illuminations (100 mW/cm^2, Model 94043A, Oriel). The spectral responses were obtained from an EQE measurement system (QEX10, PV measurement).

The water contact angles of perovskite films were measured by POWEREACH JC2000D1, i.e. one drop of water was dropped onto the top surface of perovskite films. Left and right contact angles were taken out to calculate the average angle as the final angle. As you pointed, the uncertainties of contact angle were further calculated by measuring ten selected regions to obtain ten average contact angles for each sample. The J-V characteristics of the solar cells under illumination were determined with an AAA grade Newport (9404-3A) solar simulator system under one-sun condition (AM 1.5G, 100 mW cm^{-2}, 25 °C). A certified Newport reference cell was used to calibrate the light intensity. The devices were measured in air ambient condition. The active area of the device is 0.09 cm^2. The aperture area of 0.1 cm^2 was defined using a shadow mask during the measurement to ensure the cells were completely illuminated. The precondition-light soaking is 6 s without any bias voltage.

Figures

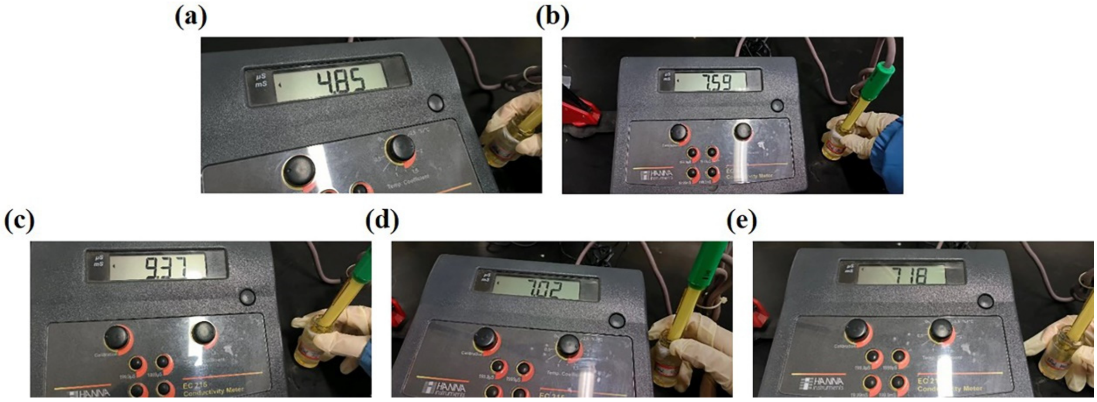

Figure S1. The photos of the conductivity tests of the (a) PC (0%)-IL, (b) PC (50%)-IL, (c) PC (70%)-IL, (d) PC (90%)-IL and (e) PC (100%)-IL composite material.

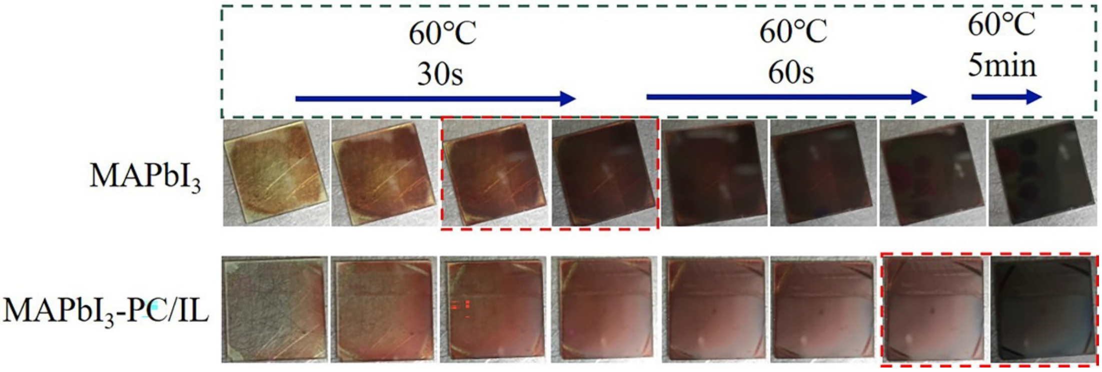

Figure S2. Photo images of the $MAPbI_3$ and $MAPbI_3$-PC/IL films during the post-annealing process under 60 ºC.

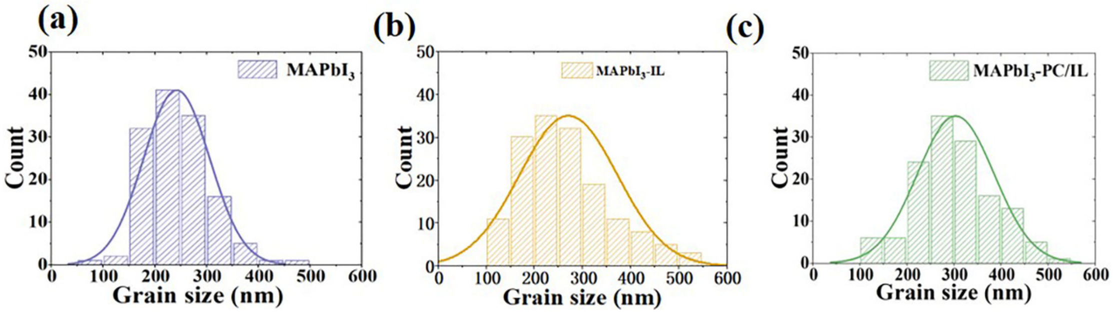

Figure S3. Histogram chart of grain size distribution of perovskite layer of (a) $MAPbI_3$ PSCs, (b)

$MAPbI_3$-IL and (c) $MAPbI_3$-PC/IL PSCs.

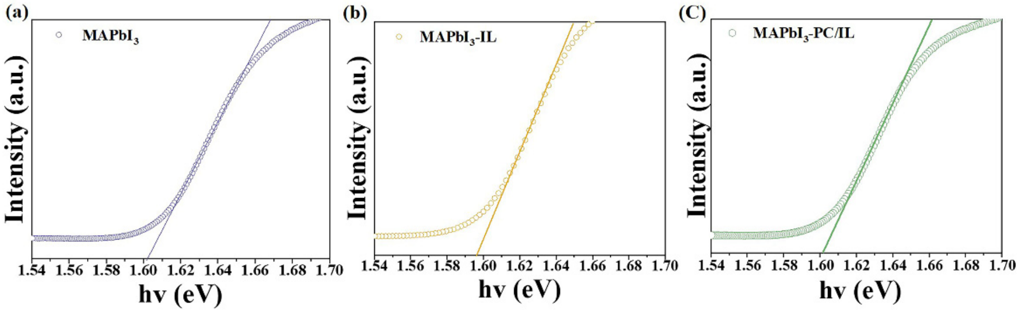

Figure S4. Tauc plots of (a) $MAPbI_3$; (b) $MAPbI_3$-IL; and (c) $MAPbI_3$-PC/IL.

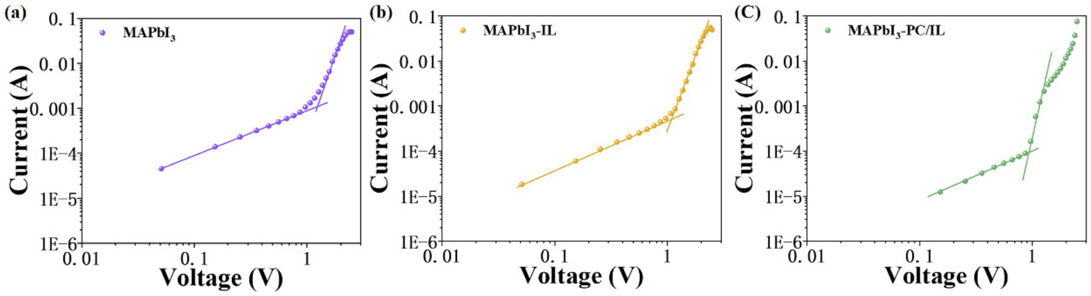

Figure S5. Dark I−V curves of devices with the structure of ITO/perovskite/Au. (a)$MAPbI_3$, (b) $MAPbI_3$-IL, (c) $MAPbI_3$-PC/IL.

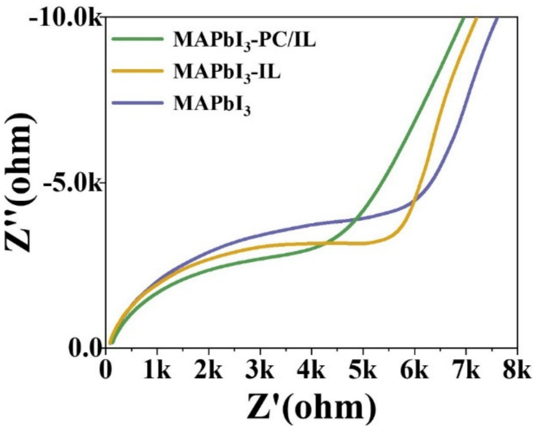

Figure S6. Nyquist plots of PSCs based on $MAPbI_3$, $MAPbI_3$-IL and $MAPbI_3$-PC/IL films in the frequency range of 1 MHz to 0.01 kHz under 0 V bias voltages.

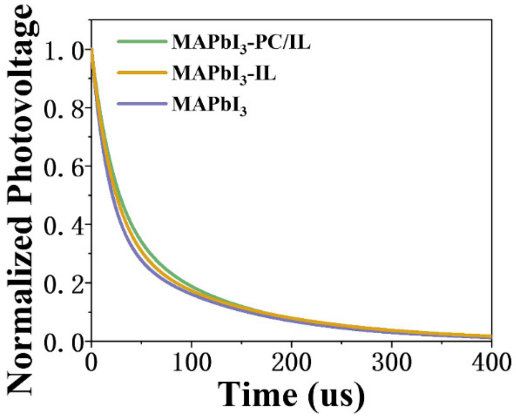

Figure S7. TPV decay curves of $MAPbI_3$, $MAPbI_3$-IL and $MAPbI_3$-PC/IL based PSCs.

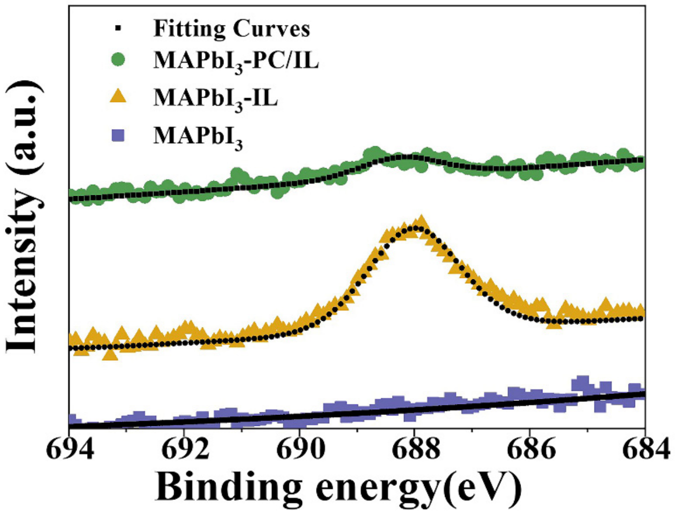

Figure S8. F1s XPS spectra of $MAPbI_3$, $MAPbI_3$-IL and $MAPbI_3$-PC/IL films.

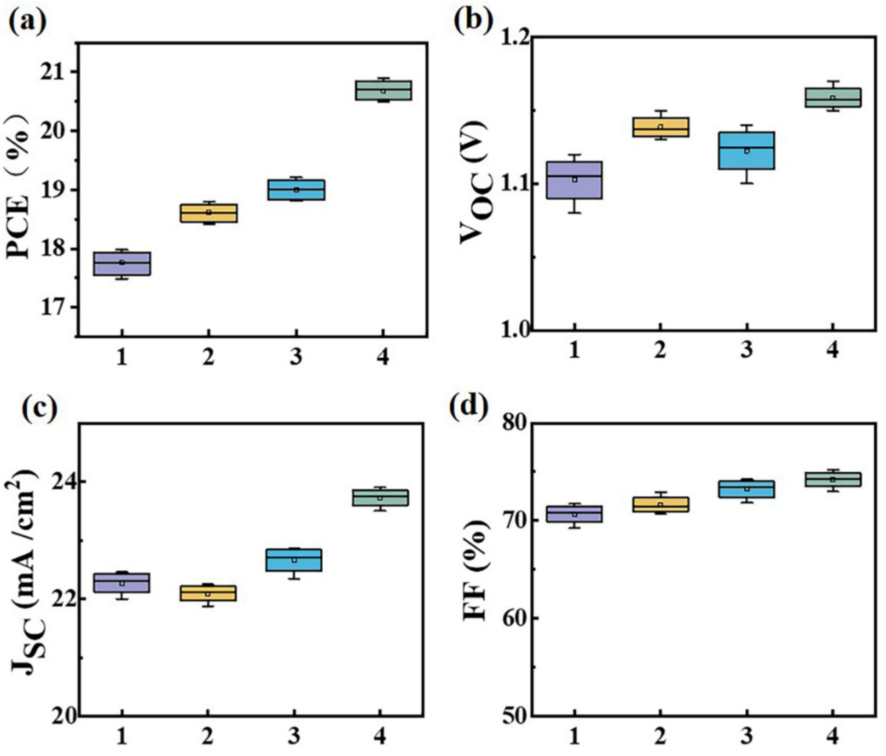

Figure S9. Statistical distributions of (a) PCE, (b) V_{OC}, (c) J_{SC}, and (d) FF for devices based on at least 10 PSCs.1, $MAPbI_3$ 2, $MAPbI_3$-IL 3, $MAPbI_3$-PC and 4, $MAPbI_3$-PC/IL.

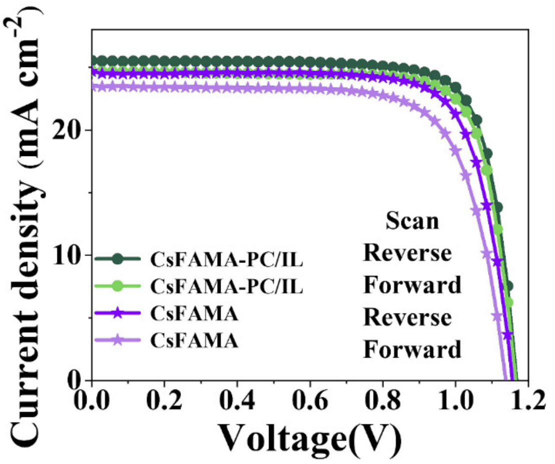

Figure S10. J-V curves of champion CsFAMA PSCs and CsFAMA-PC/IL PSCs with reverse and forward scan.

Tables

Table S1. Photovoltaic parameters of the devices shown in Figure 1d and Figure S2.

$MAPbI_3$-PC (molar fractions%)/IL	V_{OC}(V)	J_{SC} (mA cm^{-2})	FF	PCE (%)
$MAPbI_3$	1.12	22.42	71.74	17.98 (17.73±0.25)
20%	1.14	23.11	74.43	19.59 (19.38±0.21)
50%	1.15	23.43	74.65	20.10 (19.87±0.23)
70%	1.16	23.90	75.21	20.89 (20.69±0.20)
90%	1.14	22.54	72.64	18.71 (18.42±0.29)

Table S2. Fitting parameters of TRPL spectra for $MAPbI_3$, $MAPbI_3$-IL and $MAPbI_3$-PC/IL films.

	τ_1/ns	A_1/%	τ_2/ns	A_2/%	τ_{avg}/ns
$MAPbI_3$	13.07	0.638	47.85	0.363	36.55
$MAPbI_3$-IL	10.43	0.570	62.23	0.431	53.00
$MAPbI_3$-PC/IL	24.90	0.501	90.00	0.500	75.89

Table S3. Fitting parameters of the EIS spectra of $MAPbI_3$, $MAPbI_3$-IL and $MAPbI_3$-PC/IL PSCs.

Devices	R_S (Ω cm^2)	R_{ct} (Ω cm^2)	R_{rec} (Ω cm^2)
$MAPbI_3$	52.21	6101	$8{,}53\times10^4$
$MAPbI_3$-IL	57.49	6819	9.29×10^4
$MAPbI_3$-PC/IL	54.19	5202	1.02×10^5

Table S4. Photovoltaic parameters of champion $MAPbI_3$, $MAPbI_3$-IL, $MAPbI_3$-PC/IL based PSCs.

Perovskite layer	V_{OC}(V)	J_{SC} (mA cm^{-2})	FF	PCE (%)
**$MAPbI_3$**	1.12 (1.10±0.02)	22.42 (22.23±0.23)	71.74 (70.50±1.24)	17.98 (17.73±0.25)
**$MAPbI_3$-IL**	1.14 (1.14±0.01)	22.14 (21.95±0.19)	74.15 (73. 05±1.10)	18.80 (18.60±0.20)
**$MAPbI_3$-PC**	1.13 (1.12±0.02)	22.82 (22.60±0.0.26)	74.23 (73.03±1.20)	19.21 (19.01±0.21)
**$MAPbI_3$-PC/IL**	1.16 (1.16±0.01)	23.90 (23.70±0.20)	75.21 (74.11±1.10)	20.89 (20.69±0.20)

Table S5. Summary of representative results of ionic liquid additives for improving the performance of $MAPbI_3$ based PSCs.

Structure of ILs	Name of ILs	Structure of ILs	Control device PCE	Target Device PCE	Year	Ref.
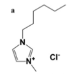	HMImCl	ITO/Compact-TiO_2/$MAPbI_3$/Spiro-OMeTAD/Ag	5.10%	2.44%	2015	S1
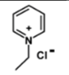	1-EC	ITO/PEDOT: PSS/$MAPbI_3$/PCBM/Al	5.06%	12.04%	2016	S2
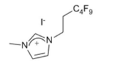	FIm	FTO/介孔 TiO_2/$MAPbI_3$/Spiro/Au	12.48%	11.55%	2016	S3
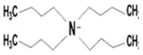	[TBAM][CL]		5.02%	5.63%		
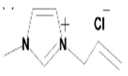	[AMIM][CL]	ITO/compact TiO_x/$MAPbI_3$ NPs/Spiro-OMeTAD/Ag	5.02%	3.93%	2017	S4
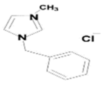	[BMIM][CL]		5.02%	3.56%		
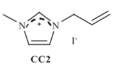	CC2		17.92%	19.14%		
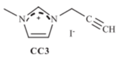	CC3		17.92%	18.55%	2017	S5
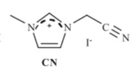	CN	FTO/c-TiO_2/meso-TiO_2/ $MAPbI_3$/Spiro-OMeTAD/Au	17.92%	16.42%		

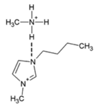	BMIBr	FTO/compact-TiO_2/TKD-TiO_2/$MAPbI_3$/Spiro-OMeTAD/Au	10.09%	10.55%	2018	S6
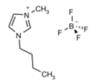	$BMIMBF_4$	FTO/NiO /$MAPbI_3$/PCBM/BCP/Cr/Cr_2O_3/Au	16%	17.8%	2018	S7
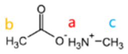	MAAc	ITO/CPTA/BACl/$MAPbI_3$/Spiro-OMeTAD/MoO_3/Au	15.23%	20.05%	2019	S8
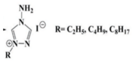	RATZ	ITO/TiO_2/$MAPbI_3$/Spiro-OMeTAD/Au	16.54%	20.31%	2019	S9
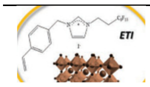	ETI	ITO/c-TiO_2/mp-TiO_2/$MAPbI_3$/Spiro-OMeTAD/Au	19.14%	19.42%	2019	S10
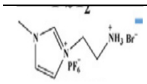	$ILPF_6$	FTO/compact-TiO_2/mesoporous-TiO_2/$MAPbI_3$/carbon	10.08%	13.01%	2019	S11
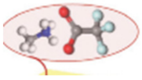	MATFA	FTO/compact-TiO_2/$MAPbI_3$/Spiro-OMeTAD/Au	17.9%	20.1%	2019	S12
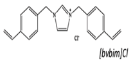	[bvbim]Cl	FTO/c-TiO_2/m-TiO_2/$MAPbI_3$/Spiro-OMeTAD/Au	15.311%	17.18%	2020	S13
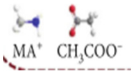	MAAc	ITO/SnO_2/$MAPbI_3$/Spiro-OMeTAD/MoO_3/Au.	20.49%	21.18%	2020	S14
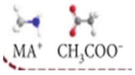	MAAc+HCl	ITO/SnO_2/BACl/ $MAPbI_3$/Spiro-OMeTAD/MoO_3/Ag	18.65%	20.78%	2021	S15
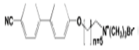	6CNBP-N	ITO/NiO_x/$MAPbI_3$/PCBM/Ag	18.07%	20.45%	2021	S16
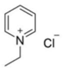	1-EC	FTO/c-TiO_2/meso-TiO_2/meso-ZnO_2/ $MAPbI_3$/Carbon	9.79%	15.14%	2021	S17
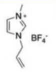	$[AMIm]BF_4$	FTO/ c-TiO_2/m-TiO_2/ZrO_2/$MAPbI_3$/carbon	13.38%	14.37%	2022	S18
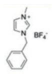	$[BzMIm]BF_4$		13.38%	16.8%		
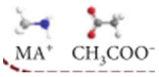	MAAc	ITO/SnO_2/$MAPbI_3$/PCBM/LiF/Al	/	19.14%	2022	S19
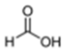	MAF	ITO/SnO_2/$MAPbI_3$/Spiro-OMeTAD/MoO_3/Ag	/	3.87%	2022	S20
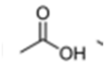	MAA			18.99%		

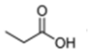	**MAP**			**20.56%**		
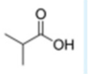	**MAIB**			**5.88%**		
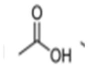	**MAAc**	**FTO/c-TiO$_2$/meso-TiO$_2$/ZnO$_2$/MAPbI$_3$/carbon**	**11.74%**	**14.82%**	**2022**	**S21**
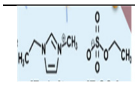	**EMIMEtSO4**					
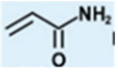	**AAM**	**ITO/PTAA/MAPbI$_3$/choline chloride/ C60 /BCP/ Cu**	**20.17%**	**21.76%**	**2022**	**S22**
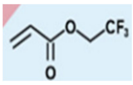	**TFEA**					
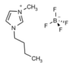	**BMIMBF$_4$**	**FTO/c-TiO$_2$/meso-TiO$_2$/(PDA)MA$_4$Pb$_5$I$_{16}$/Spiro-OMeTAD/Au**	**12.45%**	**14.07%**	**2022**	**S23**
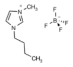	**BMIMBF$_4$**	**ITO glass/NiO$_x$/perovskite/C$_{60}$/BCP/Ag)**	**16.40%**	**17.43**	**2022**	**S34**
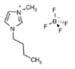	**BMIMBF$_4$** + **CQDs**	**ITO/SnO$_2$/MAPbI$_3$/Spiro-OMeTAD$_3$/Ag**	**18.14%**	**20.84%**	**2023**	**S25**
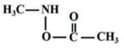 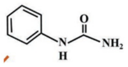	**MAAC** + **phenylurea**	**ITO/SnO$_2$/MAPbI$_3$/Spiro-OMeTAD$_3$/Ag**	**17.94%**	**20.34%**	**2023**	**S26**
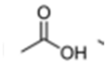	**MAAc**	**ITO/SnO$_2$/MAPbI$_3$/Spiro-OMeTAD/MoO$_3$/Ag**	**18.60%**	**20.62%**	**2023**	**S27**
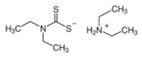	**DADA**	**ITO/PTAA/MAPbI$_3$/PCBM/BCP/Ag**	**19.13%**	**21.36%**	**2023**	**S28**
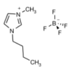 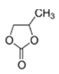	**BMIMBF$_4$** + **PC**	**ITO/SnO$_2$/MAPbI$_3$/Spiro-OMeTAD$_3$/Ag**	**17.98%**	**20.89%**	**2023**	**this work**

Table S6. The photovoltaic parameters and hysteresis index of champion $CsFAMAPbI_3$ and $CsFAMAPbI_3$-PC/IL PSCs.

**Perovskite layer**	**Scan direction**	**V_{OC}(V)**	**J_{SC} (mA cm^{-2})**	**FF (%)**	**PCE (%)**
CsFAMA	Forward	1.136	23.52	73.15	19.55
	Backward	1.154	24.58	76.15	21.61
CsFAMA-PC/IL	Forward	1.162	24.71	78.12	22.45
	Backward	1.164	25.52	78.37	23.29

Table S7. Summary of representative results of ionic liquid additives for improving the performance of CsFAMA based PSCs.

**Structure of ILs**	**Name of ILs**	**Structure of ILs**	**Control device PCE**	**Target Device PCE**	**Year**	**Ref.**
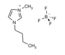	**$BMIMBF_4$**	**FTO/NiO / $(FA_{0.83}MA_{0.17})_{0.95}Cs_{0.05}Pb(I_{0.9}Br_{0.1})_3$ /PCBM/BCP/Cr/Cr_2O_3/Au**	**18.5%**	**19.8%**	**2019**	**S29**
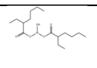	**ALEH**	**FTO/TiO_2/ $Cs_{0.05}FA_{0.95}MA_{0.05}PbI_3$/Spiro-OMeTAD/Au**	**21.31%**	**22.74%**	**2020**	**S30**
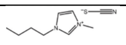 + 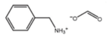	**BMIMSCN + BAFa**	**FTO/SnO_2/ $Cs_{0.05}(FA_{0.85}MA_{0.15})_{0.95}Pb(I_{0.85}Br_{0.15})_3$ /Spiro-OMeTAD/Au**	**18.90%**	**22.33%**	**2020**	**S31**
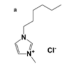	**HMImCl**	**ITO/TiO_2/ $Cs_{0.05}(FA_{0.83}MA_{0.17})_{0.95}Pb(I_{0.83}Br0_{.17})_3$/Spiro-OMeTAD/Au**	**17.33%**	**19.44%**	**2021**	**S32**
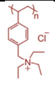	**PIL-Am**	**(ITO)/SnO_2/$Cs_{0.05}(FA_{0.85}MA_{0.15})_{0.9}5Pb(I_{0.95}Br_{0.05})_3$/Spiro-OMeTAD/Ag**	**20.26%**	**22.22%**	**2022**	**S33**
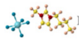	**$BMIMSbF_6$**	**FTO/SnO_2/CsFAMA-based perovskite/spiroMeOTAD/Ag**	**19.07%**	**22.03%**	**2022**	**S34**
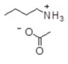	**BAAc**	**ITO/PTAA/$Cs_{0.05}(FA_xMA_y)_{0.95}Pb$ $(I_{x+0.05}Br_yCl_y)_3$/choline chloride/C_{60}/BCP/Ag**	**18.6%**	**20.2%**	**2022**	**S35**
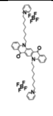	**QAPyBF4**	**FTO/SnO_2/$(CsI)_{0.05}(FAPbI_3)_{0.79}(MAPbBr_3)_{0.16}$/spiro-OMeTAD/Au**	**20.9%**	**23.1%**	**2022**	**S36**
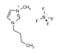 + 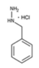 +	**$BMIMBF_4$ + BHC + OAm**	**ITO/MeO-2PACz /$(Cs_{0.05}MA_{0.14}FA_{0.81}Pb(I_{0.9}Br_{0.1})_3$ /PCBM/BCP/Cu**	**16.35%**	**20.31%**	**2023**	**S37**

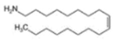						
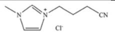	[C3CNmim]Cl	(FTO)/cp-TiO_2/mp-TiO_2/SnO_2/ ($Cs_{0.08}FA_{0.8}MA_{0.12}$) $Pb(I_{0.88}Br_{0.12})_3$/ (PEAI) (HTM)/Au	20.94%	22.86%	2022	S38
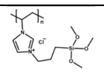	PImIL-SiO	ITO/ SnO_2/$Cs_{0.05}FA_{0.85}MA_{0.10}Pb(I_{0.97}Br_{0.03})_3$/Spiro-OMeTAD/Ag	20.68%	22.46%	2022	S39
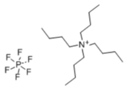	$TEAPF_6$	ITO/SnO_2/$Cs_{0.05}MA_{0.68}FA_{0.27}PbI_{3-x}Cl_x$ /Spiro-OMeTAD/Ag	20.23%	22.13%	2023	S0
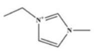	Emimcl	FTO/TiO_2/ $Cs_{0.05}(FA_{0.83}MA_{0.17})_{0.95}Pb(I_{0.83}Br_{0.17})_3$/Spiro-OMeTAD/Au	18.12%	21.54%	2023	S41
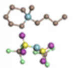	BMPTFSI	ITO/SnO_2/$Cs_{0.05}(FA_{0.83}MA_{0.17})_{0.95}$ $Pb(I_{0.9}Br_{0.1})_3$/Spiro-OMeTAD/Au	21.07%	23.34%	2023	S42
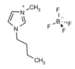 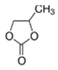	$BMIMBF_4$ + PC	ITO/SnO_2/CsFAMA/Spiro-$OMeTAD_3$/Ag	21.61%	23.29%	2023	this work

Reference

[S1] Shahiduzzaman, M.; Yamamoto, K.; Furumoto, Y.; Kuwabara, T.; Takahashi, K.; Taima, T. Ionic liquid-assisted growth of methylammonium lead iodide spherical nanoparticles by a simple spin-coating method and photovoltaic properties of perovskite solar cells. *RSC Adv.* **2015**, 5, 77495-77500.

[S2] Wan, Y.Y.; Dong, S.J.; Wang, Y.L.; Yang, L.Y.; Qin, W.J.; Cao, H.Q.; Yao, C.; Ge, Z.Y.; Yin, S.G. Ionic liquid-assisted perovskite crystal film growth for high performance planar heterojunction perovskite solar cells. *RSC Adv.* **2016**, 6, 97848-97852.

[S3] Salado, M.; Ramos, F.J.; Manzanares, V.M.; Gao, P.; Nazeeruddin, M.K.; Dyson, P.J.; Ahmad, S. Extending the Lifetime of Perovskite Solar Cells using a Perfluorinated Dopant. *ChemSusChem* **2016**, 9, 2708-2714.

[S4] Shahiduzzaman, M.; Yamamoto, K.; Furumoto, Y.; Yonezawa, K.; Hamada, K.; Kuroda, K.; Ninomiya, K.; Karakawa, M.; Kuwabara, T.; Takahashi, K.; Takahashi, K.; Taima, T. Viscosity effect of ionic liquid-assisted controlled growth of CH3NH3PbI3 nanoparticle-based planar perovskite solar cells. *Org. Electron.* **2017**, 48, 147-153.

[S5] Zhang, Y.; Fei, Z.; Gao, P.; Lee, Y.; Tirani, F.F.; Scopelliti, R.; Feng, Y.; Dyson, P.J.; Nazeeruddin, M.K. A Strategy to Produce High Efficiency, High Stability Perovskite Solar Cells Using Functionalized Ionic Liquid-Dopants. *Adv. Mater.* **2017**, 29, 1702157-1702164.

[S6] Du, J.; Wang, Y.; Zhang, Y.; Zhao, G.; Jia, Y.; Zhang, X.; Liu, Y. Ionic Liquid-Assisted Improvements in the Thermal Stability of CH3NH3PbI3 Perovskite Photovoltaics. *physica status solidi (RRL) - Rapid Research Letters* **2018**, 12.

[S7] Bai, S.; Da, P.; Li, C.; Wang, Z.; Yuan, Z.; Fu, F.; Kawecki, M.; Liu, X.; Sakai, N.; Wang, J.T.; Huettner, S.; Buecheler, S.; Fahlman, M.; Gao, F.; Snaith, H.J. Planar perovskite solar cells with long-term stability using ionic liquid additives. *Nature* **2019**, 571, 245-250.

[S8] Chao, L.F.; Xia, Y.D.; Li, B.X.; Xing, G.C.; Chen, Y.H.; Huang, W. Room-Temperature Molten Salt for Facile Fabrication of Efficient and Stable Perovskite Solar Cells in Ambient Air. *Chem* **2019**, 5, 995-1006.

[S9] Wang, S.; Li, Z.; Zhang, Y.; Liu, X.; Han, J.; Li, X.; Liu, Z.; Liu, S.; Choy, W.C.H. Water-Soluble Triazolium Ionic-Liquid-Induced Surface Self-Assembly to Enhance the Stability and Efficiency of Perovskite Solar Cells. *Adv. Funct. Mater.* **2019**, 29, 1900417-1900427.

[S10] Xia, R.; Fei, Z.; Drigo, N.; Bobbink, F.D.; Huang, Z.; Jasiūnas, R.; Franckevičius, M.; Gulbinas, V.; Mensi, M.; Fang, X.; Roldán-Carmona, C.; Nazeeruddin, M.K.; Dyson, P.J. Retarding Thermal Degradation in Hybrid Perovskites by Ionic Liquid Additives. *Adv. Funct. Mater.* **2019**, 29, 1902021-1902030.

[S11] Zhou, X.; Wang, Y.Y.; Li, C.Y.; Wu, T. Doping amino-functionalized ionic liquid in perovskite crystal for enhancing performances of hole-conductor free solar

cells with carbon electrode. *Chem. Eng. J.* **2019**, 372, 46-52.
[S12] Liu, D.; Shao, Z.; Gui, J.; Chen, M.; Liu, M.; Cui, G.; Pang, S.; Zhou, Y. A polar-hydrophobic ionic liquid induces grain growth and stabilization in halide perovskites. *Chem Commun (Camb)* **2019**, 55, 11059-11062.
[S13] Xia, R.; Gao, X.X.; Zhang, Y.; Drigo, N.; Queloz, V.I.E.; Tirani, F.F.; Scopelliti, R.; Huang, Z.; Fang, X.; Kinge, S.; Fei, Z.; Roldan-Carmona, C.; Nazeeruddin, M.K.; Dyson, P.J. An Efficient Approach to Fabricate Air-Stable Perovskite Solar Cells via Addition of a Self-Polymerizing Ionic Liquid. *Adv. Mater.* **2020**, 32, 2003801-2003810.
[S14] Chao, L.; Niu, T.; Gu, H.; Yang, Y.; Wei, Q.; Xia, Y.; Hui, W.; Zuo, S.; Zhu, Z.; Pei, C.; Li, X.; Zhang, J.; Fang, J.; Xing, G.; Li, H.; Huang, X.; Gao, X.; Ran, C.; Song, L.; Fu, L.; Chen, Y.; Huang, W. Origin of High Efficiency and Long-Term Stability in Ionic Liquid Perovskite Photovoltaic. *Research (Wash D C)* **2020**, 2020, 2616345.
[S15] Fang, M.; Tao, L.; Wu, W.; Wei, Q.; Xia, Y.D.; Li, P.; Ran, X.Q.; Zhong, Q.; Xing, G.C.; Song, L.; Muller-Buschbaum, P.; Zhang, H.; Chen, Y.H. Residual solvent extraction via chemical displacement for efficient and stable perovskite solar cells. *J. Energy Chem.* **2021**, 61, 8-14.
[S16] Xia, X.; Peng, J.; Wan, Q.; Wang, X.; Fan, Z.; Zhao, J.; Li, F. Functionalized Ionic Liquid-Crystal Additive for Perovskite Solar Cells with High Efficiency and Excellent Moisture Stability. *ACS Appl Mater Interfaces* **2021**, 13, 17677-17689.
[S17] Zhang, W.; Du, J.; Qiu, C.; Yang, K.; Huang, Q.; Wang, Q.; Zhang, W.; Han, H.; Gao, X.; Hu, Y. Enhanced efficiency of printable mesoscopic perovskite solar cells using ionic liquid additives†. *Chem Commun (Camb)* **2021**, 57, 4027-4030.
[S18] Chen, Y.; Ma, D.; Wang, Z.Q.; He, J.S.; Gong, X.Q.; Wu, W.J. Tautomeric Dual-Site Passivation for Carbon-Based Printable Mesoscopic Perovskite Solar Cells. *Adv. Mater. Interfaces* **2022**, 9, 2200326-2200337.
[S19] Chen, Y.; Xu, Y.M.; Liu, J.; Lin, Y.X.; Hu, J.F.; Cao, C.S.; Xia, Y.D.; Chen, Y.H. A universal ionic liquid solvent for non-halide lead sources in perovskite solar cells. *J. Energy Chem.* **2022**, 71, 445-451.
[S20] Gu, L.; Ran, C.; Chao, L.; Bao, Y.; Hui, W.; Wang, Y.; Chen, Y.; Gao, X.; Song, L. Designing Ionic Liquids as the Solvent for Efficient and Stable Perovskite Solar Cells. *ACS Appl. Mater. Interfaces* **2022**, 1, 21035-21044.
[S21] Jong, Y.H.; Kim, P.; Ryu, K.I.; Sonu, K.S.; Ri, J.H.; So, C.I. Effect of Ionic Liquid Methylammonium Acetate on the Performance of Hole-Transport Material-Free Mesoporous Perovskite Solar Cells with Carbon Electrode. *J. Electron. Mater.* **2022**, 51, 7085–7091.
[S22] Kang, Y.F.; Li, R.; Wang, A.R.; Kang, J.Y.; Wang, Z.S.; Bi, W.H.; Yang, Y.; Song, Y.L.; Dong, Q.F. Ionogel-perovskite matrix enabling highly efficient and stable flexible solar cells towards fully-R2R fabrication. *Energy Environ. Sci.* **2022**, 15, 3439-3448.
[S23] Ma, W.B.; Zhang, Z.L.; Kang, M.; Liu, Y.F.; Zhang, H.F.; Gao, H.P.; Mao, Y.L. Enhanced efficiency and stability of Dion-Jacobson quasi-two-dimensional perovskite solar cells by additive. *Journal of Physics D-Applied Physics* **2022**, 55, 414002-

414012.
[S24] Wang, H.Y.; Zou, W.J.; Ouyang, Y.K.; Luo, H.; Liu, X.C.; Li, H.M.; Lei, Y.; Ni, Y.F.; Fu, Y.; Zheng, D. Inducing crystal-oriented growth while inhibiting grain boundary migration with multifunctional ionic liquid for high-efficiency perovskite solar cells. *J. Alloys Compd.* **2022**, 929, 167051-167058.
[S25] Chi, S.; Yang, S.; Wang, Y.; Li, D.; Zhang, L.; Fan, L.; Wang, F.; Liu, X.; Liu, H.; Wei, M.; Yang, J.; Yang, L. Break through the Steric Hindrance of Ionic Liquids with Carbon Quantum Dots to Achieve Efficient and Stable Perovskite Solar Cells. *ACS Appl Mater Interfaces* **2023**, 15, 48304-48315.
[S26] Li, H.Y.; Feng, X.X.; Huang, K.Q.; Lu, S.Y.; Wang, X.Y.; Feng, E.R.; Chang, J.H.; Long, C.Y.; Gao, Y.J.; Chen, Z.H.; Yi, C.Y.; He, J.; Yang, J.L. Constructing Additives Synergy Strategy to Doctor-Blade Efficient $CH_3NH_3PbI_3$ Perovskite Solar Cells under a Wide Range of Humidity from 45% to 82%. *Small* **2023**, 19, 2300374-2300383.
[S27] Lv, Y.F.; Wang, K.Y.; Lan, A.; Chao, L.F.; Chen, C.S.; Wang, J.P.; Yao, Q.; Chen, J.X.; Pan, T.F.; Li, Y.J.; Zhang, H.; Xia, Y.D.; Chen, Y.H. Low-photovoltage-loss pringting perovskite solar cells in ambient air through ink synergistic engineering. *Chem. Eng. J.* **2023**, 469, 143909-143917.
[S28] Duan, S.C.; Sun, Q.; Liu, G.; Deng, J.G.; Meng, X.X.; Shen, B.; Hu, D.; Kang, B.A.; Silva, S.R.P. Synergistic Surface Defect Passivation of Ionic Liquids for Efficient and Stable $MAPbI_3$-Based Inverted Perovskite Solar Cells. *ACS Appl. Mater. Interfaces* **2023**, 15, 46483-46492.
[S29] Bai, S.; Da, P.; Li, C.; Wang, Z.; Yuan, Z.; Fu, F.; Kawecki, M.; Liu, X.; Sakai, N.; Wang, J.T.; Huettner, S.; Buecheler, S.; Fahlman, M.; Gao, F.; Snaith, H.J. Planar perovskite solar cells with long-term stability using ionic liquid additives. *Nature* **2019**, 571, 245-250.
[S30] Li, D.F.; Niu, Y.J.; Yang, Z.Q.; Li, Q.; Zhang, Z.G.; Hu, L.H. Dual-function ionic liquid enhances perovskite crystal in perovskite solar cells. *Materials Letters* **2023**, 344, 134427-134432.
[S31] Zhu, M.F.; Xia, Y.R.; Qin, L.N.; Zhang, K.Q.; Liang, J.C.; Zhao, C.; Hong, D.C.; Jiang, M.H.; Song, X.M.; Wei, J.; Zhang, P.B.; Tian, Y.X.; Jin, Z. Reducing surficial and interfacial defects by thiocyanate ionic liquid additive and ammonium formate passivator for efficient and stable perovskite solar cells. *Nano Res.* **2023**, 16, 6849-6858.
[S32] Shahiduzzaman, M.; Wang, L.; Fukaya, S.; Muslih, E.Y.; Kogo, A.; Nakano, M.; Karakawa, M.; Takahashi, K.; Tomita, K.; Nunzi, J.M.; Miyasaka, T.; Taima, T. Ionic Liquid-Assisted $MAPbI_3$ Nanoparticle-Seeded Growth for Efficient and Stable Perovskite Solar Cells. *ACS Appl Mater Interfaces* **2021**, 13, 21194-21206.
[S33] Yang, J.; Sheng, W.; Li, R.; Gong, L.; Li, Y.; Tan, L.; Lin, Q.; Chen, Y. Uncovering the Mechanism of Poly(ionic-liquid)s Multiple Inhibition of Ion Migration for Efficient and Stable Perovskite Solar Cells. *Adv. Energy Mater.* **2022**, 2103652-2103661.
[S34] Wang, H.Y.; Zou, W.J.; Ouyang, Y.K.; Luo, H.; Liu, X.C.; Li, H.M.; Lei, Y.; Ni, Y.F.; Fu, Y.; Zheng, D. Inducing crystal-oriented growth while inhibiting grain

boundary migration with multifunctional ionic liquid for high-efficiency perovskite solar cells. *J. Alloys Compd.* **2022**, 929, 167051-167058.
[S35] Ran, J.H.; Wang, H.; Deng, W.; Xie, H.P.; Gao, Y.L.; Yuan, Y.B.; Yang, Y.G.; Ning, Z.J.; Yang, B. Ionic Liquid-Tuned Crystallization for Stable and Efficient Perovskite Solar Cells. *Sol. RRL* **2022**, 2200176-2200184.
[S36] Liu, X.; Min, J.; Chen, Q.; Liu, T.; Qu, G.; Xie, P.; Xiao, H.; Liou, J.J.; Park, T.; Xu, Z.X. Synergy Effect of a pi-Conjugated Ionic Compound: Dual Interfacial Energy Level Regulation and Passivation to Promote Voc and Stability of Planar Perovskite Solar Cells. *Angew Chem Int Ed Engl* **2022**, 61, e202117303-e202117313.
[S37] Castriotta, L.A.; Calabro, E.; Di Giacomo, F.; Reddy, S.H.; Takhellambam, D.; Paci, B.; Generosi, A.; Serenelli, L.; Menchini, F.; Martini, L.; Tucci, M.; Di Carlo, A. A universal multi-additive strategy to enhance efficiency and stability in inverted perovskite solar cells. *Nano Energy* **2023**, 109, 108268-108277.
[S38] Gao, X.X.; Ding, B.; Zhang, Y.; Zhang, S.L.; Turnell-Ritson, R.C.; Kanda, H.; Abuhelaiqa, M.; Shibayama, N.; Luo, W.; Li, M.; Tirani, F.F.; Scopelliti, R.; Kinge, S.; Zuttel, A.; Zhu, D.R.; Zhang, B.; Feng, Y.Q.; Fei, Z.F.; Nazeeruddin, M.K.; Dyson, P.J. Halide exchange in the passivation of perovskite solar cells with functionalized ionic. *Cell Rep. Phys. Sci.* **2022**, 3, 100848-100862.
[S39] Gong, L.Y.; Yang, J.; Sheng, W.P.; Zhong, Y.; Su, Y.; Tan, L.C.; Chen, Y.W. Dual-Resistance of Ion Migration and Moisture Erosion via Hydrolytic Crosslinking of Siloxane Functionalized Poly(Ionic Liquids) for Efficient and Stable Perovskite Solar Cells. *Ccs Chemistry* **2022**, 5, 1202-1214.
[S40] Guo, X.; Lin, Z.H.; Cao, W.Y.; Xu, Y.M.; Wang, Q.R.; Zhang, B.Y.; Hao, Y.; Chang, J.J. Multifunctional pseudohalide-based ionic liquid doping promotes efficient and stable perovskite solar cells. *J. Mater. Chem. C* **2023**, 11, 9144-9152.
[S41] Jia, J.B.; Shi, B.B.; Dong, J.; Feng, X.; Wu, Y.Q.; Cao, B.Q. 1-ethyl-3-methylimidazolium chloride ionic liquid enhance planar triple-cation perovskite solar cell efficiency. *Materials Research Bulletin* **2023**, 166, 112345-112353.
[S42] Zhang, Z.; Si, S.L.; Luo, W.Q.; Liang, Y.Q.; Xian, Z.H.; Wen, H.X.; Huang, X.X.; Yin, T.Z.; Guo, Y.X.; Wu, H.L.; Xu, Y.; Huang, S.M. Dual Functions of Defect Passivation and Hole Transport Dopant Enabled by Piperidyl Ionic Liquid for Stable and Efficient Perovskite Solar Cells. *Sol. RRL* **2023**, 7, 2300107-2300115.
